# Perturbation of adhesion molecule-mediated chondrocyte-matrix interactions by 4-hydroxynonenal binding: implication in osteoarthritis pathogenesis

**DOI:** 10.1186/ar3173

**Published:** 2010-10-26

**Authors:** Rana El-Bikai, Mélanie Welman, Yoran Margaron, Jean-François Côté, Luke Macqueen, Michael D Buschmann, Hassan Fahmi, Qin Shi, Karim Maghni, Julio C Fernandes, Mohamed Benderdour

**Affiliations:** 1 Orthopaedic Research Laboratory, Hôpital du Sacré-Coeur de Montréal and Department of Surgery, University of Montreal, 5400 Gouin Blvd. West, Montreal, QC H4J 1C5, Canada

## Abstract

**Introduction:**

Objectives were to investigate whether interactions between human osteoarthritic chondrocytes and 4-hydroxynonenal (HNE)-modified type II collagen (Col II) affect cell phenotype and functions and to determine the protective role of carnosine (CAR) treatment in preventing these effects.

**Methods:**

Human Col II was treated with HNE at different molar ratios (MR) (1:20 to 1:200; Col II:HNE). Articular chondrocytes were seeded in HNE/Col II adduct-coated plates and incubated for 48 hours. Cell morphology was studied by phase-contrast and confocal microscopy. Adhesion molecules such as intercellular adhesion molecule-1 (ICAM-1) and α1β1 integrin at protein and mRNA levels were quantified by Western blotting, flow cytometry and real-time reverse transcription-polymerase chain reaction. Cell death, caspases activity, prostaglandin E2 (PGE_2_), metalloproteinase-13 (MMP-13), mitogen-activated protein kinases (MAPKs) and nuclear factor-kappa B (NF-κB) were assessed by commercial kits. Col II, cyclooxygenase-2 (COX-2), MAPK, NF-κB-p65 levels were analyzed by Western blotting. The formation of α1β1 integrin-focal adhesion kinase (FAK) complex was revealed by immunoprecipitation.

**Results:**

Col II modification by HNE at MR approximately 1:20, strongly induced ICAM-1, α1β1 integrin and MMP-13 expression as well as extracellular signal-regulated kinases 1 and 2 (ERK^1/2^) and NF-κB-p65 phosphorylation without impacting cell adhesion and viability or Col II expression. However, Col II modification with HNE at MR approximately 1:200, altered chondrocyte adhesion by evoking cell death and caspase-3 activity. It inhibited α1β1 integrin and Col II expression as well as ERK^1/2 ^and NF-κB-p65 phosphorylation, but, in contrast, markedly elicited PGE_2 _release, COX-2 expression and p38 MAPK phosphorylation. Immunoprecipitation assay revealed the involvement of FAK in cell-matrix interactions through the formation of α1β1 integrin-FAK complex. Moreover, the modification of Col II by HNE at a 1:20 or approximately 1:200 MR affects parameters of the cell shape. All these effects were prevented by CAR, an HNE-trapping drug.

**Conclusions:**

Our novel findings indicate that HNE-binding to Col II results in multiple abnormalities of chondrocyte phenotype and function, suggesting its contribution in osteoarthritis development. CAR was shown to be an efficient HNE-snaring agent capable of counteracting these outcomes.

## Introduction

Articular cartilage is composed of chondrocytes embedded in an exquisitely-organized extracellular matrix (ECM) of collagen and proteoglycans. Chondrocytes, responsible for regenerating and maintaining cartilage, exist in a relatively-isolated environment, since this tissue lacks blood vessels, lymphatic structures and nerves [1]. The ECM is an important pleiotropic regulator of several fundamental cellular processes, such as migration, proliferation, and phenotypic expression [2]. Chondrocyte phenotype and function are partially controlled by their interactions with the surrounding ECM. These regulatory effects of ECM are mediated through cell surface "adhesion receptors" that support the attachment of cells to ECM molecules both *in vivo *and *in vitro*. At the cell surface, matrix receptors link the ECM to the cell interior through elements of the cytoskeleton and other component proteins of signal transduction pathways. In chondrocytes, the best-described families of cell surface receptors are the integrins as well as non-integrin receptors, including CD44 of the hyaluronan-binding protein family, annexin V, and intercellular adhesion molecule-1 (ICAM-1) [3,4]. Integrin attachment stimulates the formation of focal adhesion complexes, intracellular protein complexes that transduce signals from the ECM to intracellular effectors, such as the cytoskeleton [5]. Such receptors convey information from the ECM to the intracellular compartment, utilizing several signal transduction pathways. The binding of matrix components to cell surface receptors also establishes a pericellular pool of ECM molecules that may stabilize cell phenotype.

Osteoarthritis (OA), a chronic disorder affecting the elderly, is characterized by joint pain and disability. Deterioration of articular cartilage is a hallmark of OA pathogenesis [6]. Although the precise aetiology of this disease is still unknown, it is clear that the degradation of articular cartilage is mediated by various factors, such as the production of metalloproteinases (MMPs) and other products resulting from cellular activity that selectively impact the cartilage matrix [7,8]. Among them, lipid peroxidation (LPO) end-products, such as 4-hydroxynonenal (HNE) and malondialdehyde (MDA), are believed to play key roles in cartilage damage in OA [9]. Our previous study demonstrated, for the first time, that HNE level was significantly higher in synovial fluids and osteoblasts from OA patients than in normal subjects. We also reported that free HNE was capable of inducing catabolic and inflammatory responses in isolated OA chondrocytes and altering the cellular phenotype of OA osteoblasts. These responses were mediated by the modulation of a panoply of signalling pathways, including mitogen-activated protein kinases (MAPKs) and nuclear factor-kappa B (NF-κB) [10-12]. Generally, free HNE most likely represents one of the main LPO products that can modulate physiological as well as pathological processes, as depicted in a recent, dedicated review [13].

Furthermore, the relevance of LPO products to OA pathogenesis was manifested by their ability to form adducts. By binding to proteins, HNE is capable of activating MMP-13 and increasing the susceptibility of type II collagen (Col II) to proteolytic cleavage by MMP-13 [10]. The formation of HNE/Col II was augmented in human cartilage treated with tumour necrosis factor-alpha (TNFα and free radical donors. Tiku *et al. *reported that chondrocyte-derived LPO products mediate the oxidation of cartilage collagens [14]. They proposed that oxidative modification of cartilage collagen by aldehyde *in vivo *could result in alteration of the biochemical and biophysical properties of cartilage collagen fibrils, making them prone to degradation and initiating the changes observed in aging and OA.

The present study was undertaken to clarify the significance of high levels of HNE-Col II adducts in OA cartilage. We established that interactions between OA chondrocytes and HNE-modified ECM protein (for example, Col II) induced changes in cell phenotype and function, consequently contributing to cartilage damage seen during OA development. The beneficial effect of carnosine (CAR), an HNE-trapping drug, was also investigated.

## Materials and methods

### Specimen selection and chondrocyte culture

Post-surgery discarded human OA articular cartilage was obtained from OA patients (aged 67 ± 9 years mean ± SD, *n *= 27) who underwent total knee arthroplasty. Informed consent had been obtained from patients with OA for the use of their tissues for research purposes. All patients were evaluated by rheumatologists who followed American College of Rheumatology criteria [15]. The experimental protocols and use of human tissues were approved by the Research Ethics Board of Hôpital du Sacré-Cœur de Montréal.

OA knee cartilage specimens were spliced and rinsed, and chondrocytes were extracted by sequential enzymatic digestion, as described previously [10]. Cartilage samples were digested with 1 mg/ml of pronase (Sigma-Aldrich, Oakville, ON, Canada) for one hour at 37°C, followed by 2 mg/ml of type IV collagenase (Sigma-Aldrich) for six hours in Dulbecco's modified Eagle's medium (Invitrogen, Burlington, ON, Canada) supplemented with 10% heat-inactivated fetal bovine serum (FBS, Invitrogen), 100 units/ml of penicillin and 100 μg/ml of streptomycin (Invitrogen). The cells were seeded at high density in culture flasks at 37°C in a humidified atmosphere of 5% CO_2_/95% air until they were confluent and ready for experimentation.

### Plate coating and cell seeding

Human Col II (Sigma-Aldrich) was modified with HNE (Cayman Chemical Company, Ann Arbor, Ml, USA) at different molar ratios (MR) (1:20 to 1:200; Col II:HNE). 24-well plates (for cell suspensions) were coated with 0.1 mg/ml HNE-Col II adducts and incubated for 24 h at 4°C. They were washed three times with phosphate-buffered saline (PBS) to eliminate free HNE and conserved at 4°C until used. First-passage chondrocytes were distributed into pre-treated 24-well plates, as outlined above, at concentrations of 2 × 10^5 ^cells/cm^2 ^in 1 ml in DMEM containing 2% FBS, and incubated at 37°C for 48 h in a humidified atmosphere containing 5% CO_2_/95% air.

### Cell morphology and viability

Changes in cell morphology were studied by phase-contrast microscopy with a ×20/40 objective after hematoxylin and eosin staining (*n *= 5). Chondrocyte viability was evaluated as described previously [16] using 3-(4,5-dimethyl-thiazoyl)-2,5-diphenyl-SH-tetrazolium bromide (MTT) assay in 96-well plates (Fisher Scientific Company, Ottawa, ON, Canada) by incubating them with 0.5 mg/ml MTT reagent (Sigma-Aldrich) for 15 minutes at 37°C. Then, 100 μL of solubilization solution (0.04 M HCl-isopropanol) was added, formazan salt was dissolved, and absorbance was read at 570 nm with the micro-ELISA Vmax photometer (Bio-Tek Instruments, Winooski, VT, USA).

### Cell spreading assays

Chondrocytes were grown for 48 h in eight-well LabTeck chambers (2 × 10^5 ^cells/cm^2^) coated with 0.1 mg/ml HNE-Col II adducts. Cells were fixed by 4% paraformaldehyde for 30 minutes. An immunofluorescence was performed and the areas, perimeters, Feret's diameters and circularity index of more than 200 cells were analyzed using the threshold function of Image-J software (NIH) (*n *= 4 for each condition).

### Immunofluorescence

Cells were fixed with 4% paraformaldehyde, permeabilized with 0.2% Triton X-100 (Sigma-Aldrich) in PBS and blocked in PBS-1% BSA (United States Biological, Swampscott, MA, USA), and then incubated with Alexa Fluor 488 Phalloidin (1:200 dilution, Invitrogen) for 30 minutes. After one wash in Tween 0.2% (Sigma-Aldrich) in PBS and three in PBS alone, the chambers slides were mounted with coverslips using *Slow Fade^® ^*Gold antifade reagent with DAPI mounting medium (Invitrogen). Fluorescence images were captured with a Zeiss LSM710 confocal microscope, and the quantitative cell morphology analysis was performed using images taken with a Leica DM6000 epifluorescence microscope (Deerfield, IL, USA) equipped with a Retiga EXi (QImaging, Burnaby, BC, Canada) camera (*n *= 4).

### Immunoprecipitation

To demonstrate the involvement of FAK in cell-collagen interaction, OA chondrocytes (approximately 10^6 ^cells, *n *= 3) were incubated for 48 h in HNE/Col II adducts-coated plates. Then, cells were lysed on ice in 1 ml lysis buffer (KLB: 40 mM Tris (pH 8.0), 250 mM NaCl, 0.1% Nonidet P-40, 5 mM EDTA, 5 mM EGTA, 10 mM β-glycerophosphate, 10 mM NaF, 0.3 mM Na_3_VO_4_, 1 mM DTT) supplemented with protease inhibitors cocktail as described previously [11]. A total of 100 μg of total protein was subjected to immunoprecipitation with 1 μg of mouse anti-human α1β1 integrin (Santa Cruz Biotechnology, Santa Cruz, CA, USA) in KLB buffer containing 0.5 M NaCl for overnight at 4°C and then for an additional two hours with protein A (Santa Cruz Biotechnology). The resin was washed with KLB buffer and proteins were removed from the resin by the addition of 50 μl undiluted SDS-loading buffer. The immunoprecipitates were analyzed by Western blotting using mouse anti-human α1β1 integrin (Santa Cruz Biotechnology) or rabbit anti-pFAK antibody (Millipore, Etobicoke, ON, Canada), as primary antibody.

### Measurement of caspase activities

Enzymatic caspase-3/8 activities were measured with commercial kits. To measure caspase-8 activity, the cells (*n *= 5) were washed with PBS and resuspended in 100 μL of lysis buffer (R&D Systems, Minneapolis, MN, USA), left on ice for 10 minutes, and centrifuged. Protein concentration of the supernatants was measured according to the bicinchoninic acid method (Pierce, Rockford, IL, USA). Total proteins (50 μg) were reacted with 200 μM IETD-*p*NA substrate in the presence of 100 μL of reaction buffer. To quantitate caspase-3 activity, the cells were washed with PBS and lysed in 100 μL of lysis buffer (Sigma-Aldrich), left on ice for 15 minutes, and centrifuged. Total proteins (5 μg) were reacted with 200 μM DEVD-*p*NA substrate in the presence of 100 μL of reaction buffer. After 16 hours of incubation at 37°C, *p*-nitroanilide release was measured at 405 nm for caspase-3 and -8.

### Protein detection by Western blotting

A sum of 20 μg of total proteins from chondrocyte lysates (*n *= 4) treated under the indicated conditions was loaded for discontinuous 4 to 12% sodium dodecyl sulfate-polyacrylamide gel electrophoresis. They were then transferred electrophoretically onto nitrocellulose membranes (Bio-Rad Laboratories, Mississauga, ON, Canada) for protein immunodetection and semi-quantitative measurement [10]. The primary antibodies used were mouse anti-human ICAM-1, anti-human α1β1 integrin and anti-human β-actin (Santa Cruz Biotechnology), anti-Col II (Oncogene Research Products, La Jolla, CA, USA) or rabbit anti-human COX-2 (Cayman Chemical Company), anti-pFAK (Millipore), anti-total and phosphorylated extracellular signal-regulated kinases 1 and 2 (ERK^1/2^), p38 MAPK, and NF-κB-p65 (Cell Signaling Technology, Inc., Danvers, MA, USA). After serial washes, the primary antibodies were revealed by goat anti-mouse or anti-rabbit IgG conjugated to horseradish peroxidase (Cell Signaling Technology). Immunoreactive proteins were detected with SuperSignal blotting substrate (Pierce) and exposed to Kodak X-Omat film (Eastman Kodak Company, Rochester, NY, USA).

### Prostaglandin E2 (PGE_2_) and MMP-13 determination

After incubating the chondrocytes for 48 h in collagen-coated plates, the medium was collected, and PGE_2 _and MMP-13 levels were assessed by enzyme immunoassay (Cayman Chemical Company) and ELISA kits (R&D Systems). Detection sensitivity was 9 and 8 pg/ml, respectively. All assays were performed in duplicate (*n *= 6). Absorbance was measured with the micro-ELISA Vmax photometer (Bio-Tek Instruments).

### RNA extraction and reverse transcription-polymerase chain reaction (RT-PCR)

Total RNA was isolated with TRIzol reagent according to the manufacturer's instructions (Invitrogen). RNA was quantitated with RiboGreen RNA quantitation kits (Molecular Probes, Eugene, OR, USA), dissolved in diethylpyrocarbonate-treated H_2_O, and stored at -80°C until used. One μg of total RNA was reverse-transcribed with Moloney murine leukemia virus reverse transcriptase (Fermentas, Burlington, ON, Canada), as detailed in the manufacturer's guidelines. One-fiftieth of the reverse transcriptase reaction product was analyzed by real-time quantitative PCR. The following specific sense and antisense primers were purchased from Bio-Corp Inc. (Montreal, QC, Canada): human ICAM-1, 5'-CCT ATG GCA ACG ACT CCT TC-3' (forward) and 5'-TGC GGT CAC ACT GAC TGA G-3' (reverse); human α1 integrin, 5'-GGA GCA ATT CGA CGA GCA CT-3' (forward) and 5'-TTC ATC CCG CAG ATA CGC TA-3' (reverse); human β1 integrin, 5'-TTC AAT GCC ACC TGC CTC AA-3' (forward) and 5'-TTG GCC TCA ATG CTG AAG CTC-3' (reverse); human GAPDH, 5'-CAG AAC ATC ATC CCT GCC TCT-3' (forward) and 5'GCT TGA CAA AGT GGT CGT TGA G-3' (reverse); human MMP-13, 5'-CTT AGA GGT GAC TGG CAA AC (forward) and 5'-GCC CAT CAA ATG GGT AGA AG (reverse); human COX-2, 5'-TTC AAA TGA GAT TGT GGG AAA ATT GCT-3' (forward) and 5'-AGT TCA TCT CTG CCT GAG TAT CTT-3' (reverse).

Quantitative PCR analysis was performed in a total volume of 50 μl containing template DNA, 200 nM sense and antisense primers, 25 μl of SYBR Green Master Mix (Qiagen, Mississauga, Ontario, Canada), and 0.5 units of uracil-*N*-glycosylase (UNG, Epicentre Technologies, Madison, WI, USA). After incubation at 50°C for two minutes (UNG reaction) and at 95°C for 10 minutes (UNG inactivation and activation of AmpliTaq Gold enzyme), the mixtures were subjected to 40 amplification cycles (15 s at 95°C for denaturation and one minute at 60°C for annealing and extension). Incorporation of SYBR Green dye into the PCR products was monitored in real-time with a Mx3000 real-time PCR system (Stratagen, La Jolla, CA, USA), allowing determination of the threshold cycle (C_t_) at which exponential amplification of PCR products begins. After PCR, dissociation curves were generated with one peak, indicating amplification specificity. A C_t _value was obtained from each amplification curve with software provided by the manufacturer (Stratagen).

Relative mRNA expression in chondrocytes was quantified according to the ΔΔC_t _method, as detailed in the manufacturer's (Stratagen's) guidelines. A ΔC_t _value was calculated, first by subtracting the C_t _value for the housekeeping gene GAPDH from the C_t _value for each sample. A ΔΔC_t _value was then calculated by subtracting the ΔC_t _value for the control (unstimulated cells) from the ΔC_t _value for each treatment. Fold changes compared to the controls were then determined by 2^-ΔΔC^_t_. Each PCR generated only the expected specific amplicon, as shown by melting temperature profiles of the final product and by gel electrophoresis of the test PCRs. Each PCR was run in triplicate on two separate occasions for each independent experiment (*n *= 6).

### Flow cytometric analysis

Incubated cells in unmodified or HNE-modified Col II were trypsinized, centrifuged and then labeled with either 1 μg of a purified mouse IgG_1 _isotype control (BD Biosciences PharMingen; San Diego, CA, USA), or mouse anti-human ICAM-1 (15.2) (Santa Cruz Biotechnology) in PBS/2% FBS for 60 minutes at 4°C. The cells were then washed twice in PBS and incubated with Alexa Fluor^® ^488 goat anti-mouse IgG (Invitrogen) diluted 1/500 in PBS/2% FCS, for 30 minutes at 4°C in the dark. They were washed, re-suspended in 500 μl of PBS and analyzed. ICAM-1 surface expression on chondrocytes (*n *= 4) was assessed in a Coulter Epics XL flow cytometer with Expo32 software (Beckman Coulter System, Mississauga, ON, Canada).

### MAPK and NF-κB quantification

After 48 h of incubation, human OA chondrocytes (*n *= 4) were lysed, and protein levels were quantitated according to the BCA method. The samples were assayed with ELISA kits for total and phosphorylated p38 MAPK, ERK^1 ^(p44), ERK^2 ^(p42), and NF-κB-p65 (Cell Signaling Technology, Inc.). The data are expressed as fold induction.

### Statistical analysis

Results were expressed as the mean ± SEM. Statistical differences between two groups of data were analyzed using ANOVA test and Bonferroni's multiple comparison procedures. A difference of less than or equal to 0.05 was considered significant.

## Results

### HNE-binding to Coll II induces changes in cell morphology, viability and phenotype

**Figure 1:**
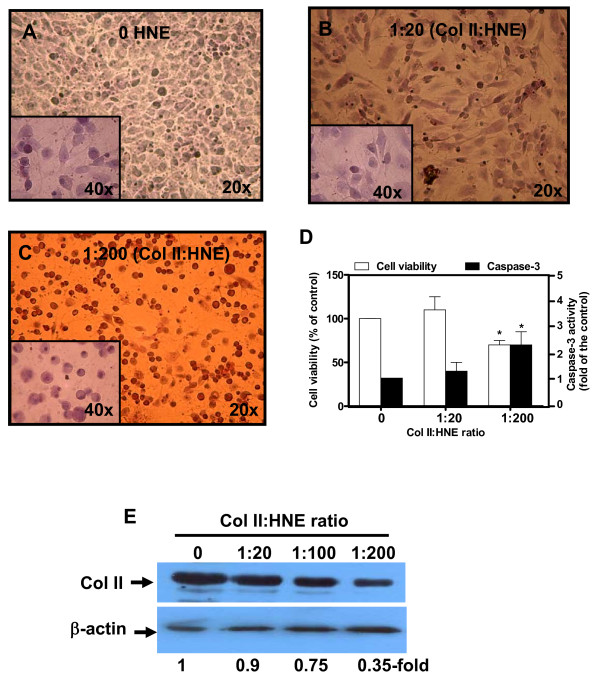
**HNE-binding to Coll II induces changes in chondrocytes morphology, viability and phenotype**. Twenty-four-well plates were coated with 0.1 mg/ml human Col II and treated after with HNE at different molar ratios (1:20 to 1:200; Col II:HNE). After several washes, human OA chondrocytes were seeded at 2 × 10^5 ^cells/cm^2 ^and incubated up to 48 h. Cell morphology of cultured chondrocytes in untreated Col II **(a)**, HNE-treated Col II at a 1:20 MR **(b)**, and HNE-treated Col II at a 1:200 MR **(c) **was observed by phase contrast microscopy after hematoxylin and eosin staining (mag. 20 to 40×). **(d) **Cell viability and caspase-3 activity were determined with MTT assay and commercial kit respectively. **(e) **Col II protein expression was analyzed in cellular extract by Western blot. ANOVA tests and Bonferroni's multiple comparison were performed to compare each condition. The data are means ± SEM of *n *= 5. * *P *< 0.05; **, *P *< 0.01.

To investigate the effect of HNE-binding to Col II on chondrocyte function, we first documented changes in cell morphology and viability by phase-contrast microscopy and MTT assay, respectively. After 48 h of incubation, Col II modification by HNE at high MR (1:200) (Figure 1c) induced alterations in cell morphology compared to unmodified Col II or collagen exposed to HNE at low MR (1:20) (Figure 1a, b). The changes in cell morphology were associated with cell viability decreased by 30% and caspase-3 activity increased by approximately two-fold respectively (Figure 1d). In the same pattern, caspase-8 activity was also increased (data not shown). Thereafter, we evaluated the ability of HNE-modified Col II to affect chondrocyte phenotype via Col II protein expression. As illustrated in Figure 1e, Col II protein expression was reduced by 25 and 65% when the cells were seeded in Coll II treated with HNE at a 1:100 MR and 1:200 MR respectively, indicating altered cell phenotype. Col II modification with HNE up to 1:100 MR did not influence cell viability, and caspase-3 activity. These data indicate that interactions between human OA chondrocytes and HNE-Coll II adduct influence cell morphology, viability and phenotype.

**Figure 2:**
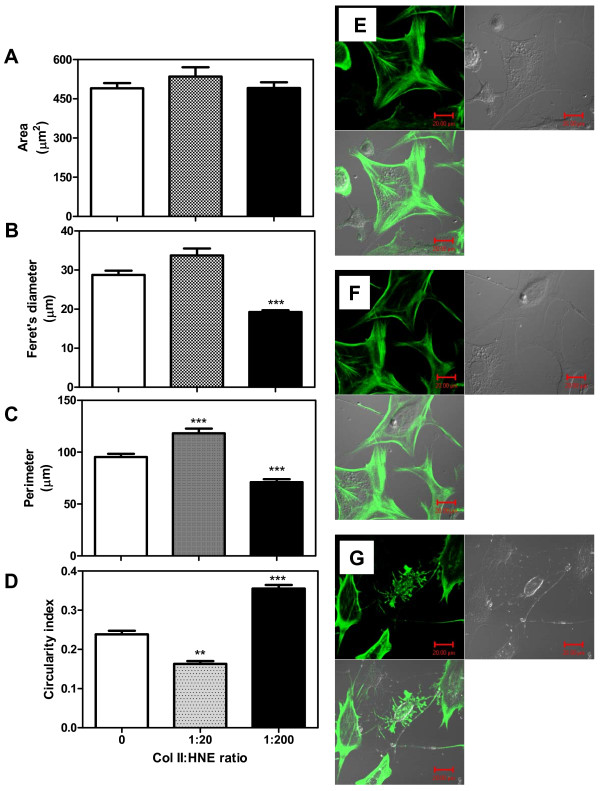
**Chondrocyte morphology analysis and actin cytoskeleton**. Chondrocytes were incubated as described in Legend 1 and cell morphology was analyzed by fluorescence microscopy using a phalloidin staining and ImageJ analysis. For each condition, the area **(a)**, the Feret's diameter **(b)**, the perimeter **(c) **and the circularity index **(d) **of more than 200 cells was measured. Cell cytoskeleton was observed by phalloidin in cultured chondrocytes in untreated Col II **(e)**, HNE-treated Col II at a 1:20 MR **(f)**, and HNE-treated Col II at a 1:200 MR **(g)**. Scale bar, 20 μm. ANOVA tests and Bonferroni's multiple comparison were performed to compare each condition. The data are means ± SEM of *n *= 4. ** *P *< 0.01; *** *P *< 0.001.

Then, chondrocytes morphology were analysed by measuring main parameters of the cell shape, including: the area, Feret's diameter, perimeter, and circularity index. Figure 2a indicates that culture conditions don't affect cell area. However, Col II modification with HNE at a 1:200 MR significantly decreased the Feret's diameter of cell compared to the control. (Figure 2b), indicating that cells make less straight membrane protrusions. Moreover, cell perimeter is significantly increased when cells were cultured in the presence of HNE-modified Col II at a 1:20 MR compared to the control (Figure 2c), but decreased in the presence of HNE-modified Col II at a 1:200 MR. In the same way, the circularity index of the cells is reduced in the presence of HNE-modified Col II at a 1:20 MR and increased then after in the presence of HNE-modified Col II at a 1:200 MR (Figure 2d). Finally, cells seem to be more spread in unmodified Col II or HNE-modified Col II at a 1:20 MR than in HNE-modified Col II at a 1:200 MR (Figure 2e-g). Taken together, these results indicate that cells are more rounded and smooth when Col II was high alkylated by HNE when compared to unmodified Col II and low alkylated Col II.

### HNE-binding to Col II modulates the expression of adhesion molecules (ICAM-1 and α1β1 integrin)

**Figure 3:**
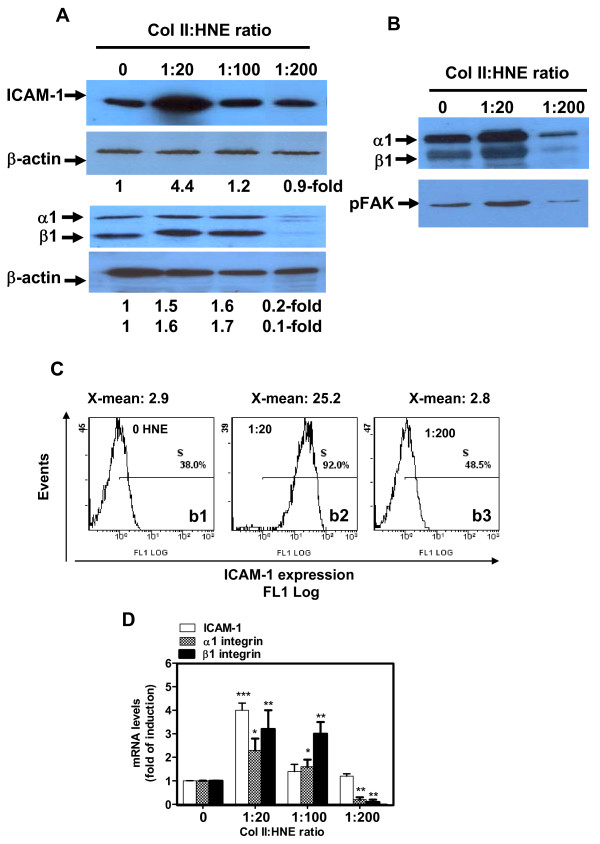
**HNE-modified Col II modulates adhesion molecules expression and focal adhesion kinase in human OA chondrocytes**. Cells were incubated as described in Legend 1 and protein **(a) **and mRNA **(b) **levels of ICAM-1 and α1β1 integrin were analyzed in cellular extracts by Western blot and real-time RT-PCR, respectively. **(b) **α1β1 integrin antibody coimmunoprecipitates pFAK. One hundred micrograms of total protein extracts of chondrocytes were immunoprecipitated with mouse anti-α1β1 integrin antibody. α1β1 immunoprecipitates were immunoblotted with pFAK or α1β1 integrin antibody. **(c) **Protein expression of ICAM-1 was qualitatively evaluated by flow cytometry using mouse anti-human ICAM-1 and Alexa FluoR 488 goat anti-mouse IgG. Mouse isotype IgG1 was used as a negative control. (b1) untreated Col II, (b2) HNE-treated Col II at a 1:20 MR, (b3) HNE-treated Col II at a 1:200 MR. ANOVA tests and Bonferroni's multiple comparison were performed to compare each condition. The data are means ± SEM of *n *= 5. *P < 0.05, **P < 0.01, ***P < 0.001.

Cell adhesion molecules are expressed on the cell surface and are involved in binding with other cells or with the ECM components. They play a critical role in a wide array of biological processes that include differentiation, viability, inflammation and catabolism. In the present study, we investigated both ICAM-1 and α1β1 integrin in cultured chondrocytes in HNE-modified Col II-coated plates. Compared to unmodified Col II, Col II modification with HNE at low MR (1:20) induced ICAM-1 expression at the protein (Figure 3a) and mRNA (Figure 3d) levels by 440 (*P *< 0.001) and 400% (*P *< 0.001), respectively. No change in ICAM-1 expression was observed when Col II was modified by HNE at a 1:100 or 1:200 MR.

We then undertook flow cytometric analysis to confirm the Western blotting and real-time PCR data. Figure 3c discloses that Col II modification by HNE at a 1:20 MR (Figure 3c.b2) increased the percentage of cells expressing ICAM-1 by two-fold at the surface of chondrocytes, compared to control cells (Figure 3c.b1). When the data were analyzed for X-mean fluorescence intensity, almost a 10-fold increment in ICAM-1 expression was noted (Figure 3c.b2). However, incubated cells with HNE-treated Col II at a 1:200 MR presented ICAM-1 expression levels comparable to those in control cells in terms of both cell percentage and X-mean fluorescence intensity (Figure 3c.b3). Flow cytometry of ICAM-1 expression on chondrocytes in response to their incubation in HNE-modified Col II was in agreement with the Western blotting and real-time PCR data.

Furthermore, α1 and β1 integrin expression increased, in the same pattern, by 150 (*P *< 0.05) and 160% (*P *< 0.05) at the protein level (Figure 3a) and by 230 (*P *< 0.05) and 310% (*P *< 0.01) at the mRNA level (Figure 3d) when Col II was modified by HNE at a 1:20 MR. However, Col II modification with HNE at a 1:200 MR dramatically decreased the expression of both α1 and β1 integrin by approximately 85% (*P *< 0.001) at the protein and mRNA levels (Figure 3a, d). Collectively, these data suggest that inhibition of α1β1 integrin expression will be involved in alterations of chondrocyte morphology, viability and phenotype, as observed in Figure 1.

Coimmunoprecipitation experiments were performed to determine the involvement of FAK in cell-matrix interactions. Our data showed that pFAK is detected in α1β1 integrin immunoprecipitates from cultured chondrocytes (Figure 3b). The phosphorylated level of FAK increased when Col II was modified by HNE at a 1:20 MR and then decreased when Col II was modified by HNE at a 1:200 MR.

### Interactions between HNE-modified Col II and chondrocytes induce inflammatory and catabolic responses

**Figure 4:**
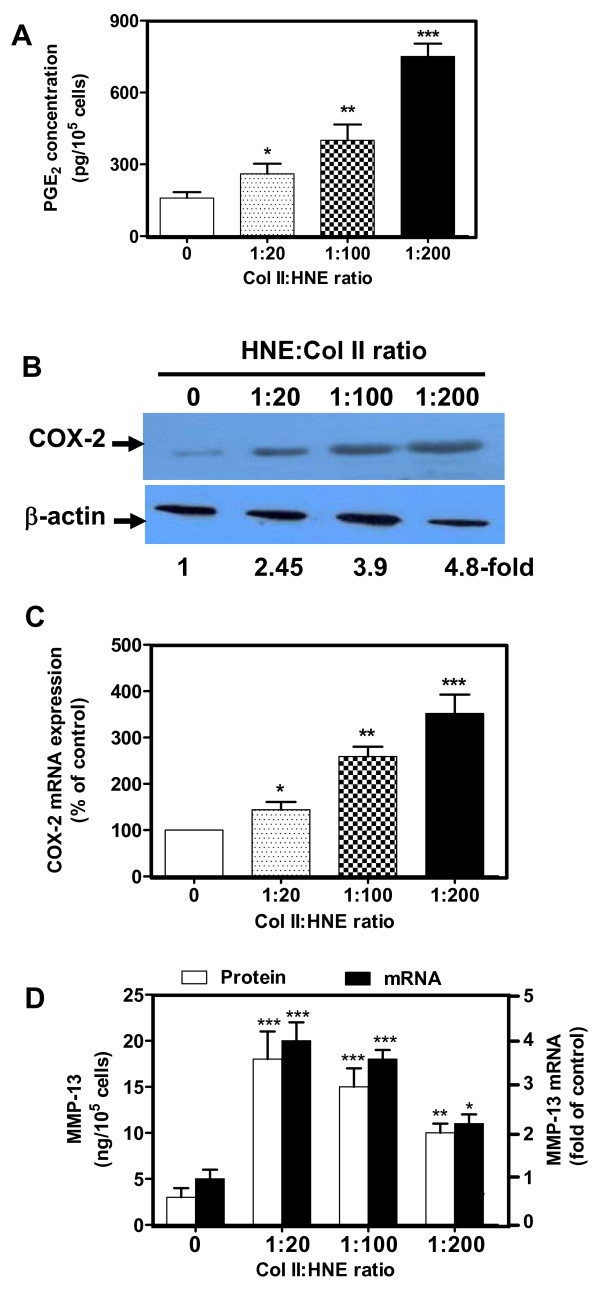
**Interactions between human OA chondrocytes and HNE-modified Col II induced COX-2 and MMP-13 expression**. Cells were incubated as described in Legend 1 and culture media was collected. PGE_2 _**(a) **and MMP-13 **(d) **release was assessed by enzyme immunoassay and ELISA kits. Levels of COX-2 protein **(b) **as well as COX-2 mRNA **(c) **and MMP-13 mRNA (d) were analyzed in cellular extracts by Western blot and real-time RT-PCR, respectively. ANOVA tests and Bonferroni's multiple comparison were performed to compare each condition. The data are means ± SEM of *n *= 6. *P < 0.05, **P < 0.01, ***P < 0.001.

In the next series of experiments, we investigated whether interactions between chondrocytes and their surrounding matrix, HNE-modified Col II, regulate the production of factors known to be involved in OA, such as PGE_2, _a product of COX-2, and MMP-13. Our data revealed that HNE-modified Col II induced PGE_2 _release and COX-2 expression. PGE_2 _(Figure 4a) peaked at 720 pg/ml (*P *< 0.001), and COX-2 protein and mRNA (Figure 4b, c) reached 480 and 350% (*P *< 0.001), respectively, when Col II was treated with HNE at a 1:200 MR. We additionally established that HNE-modified Col II evoked a significant increase of MMP-13 but inversely to COX-2. MMP-13 protein and mRNA (Figure 4d) reached maximum levels of 17 ng/ml (*P *< 0.001) and 400% (*P *< 0.001), respectively, when Col II was modified with HNE at a 1:20 MR. Taken together, these findings suggest that interactions between chondrocytes and HNE-modified Col II may contribute to the production of inflammatory and catabolic mediators known to be involved in OA.

### HNE-binding to Col II activates p38 MAPK, ERK^1/2 ^and NF-κB-p65

**Figure 5:**
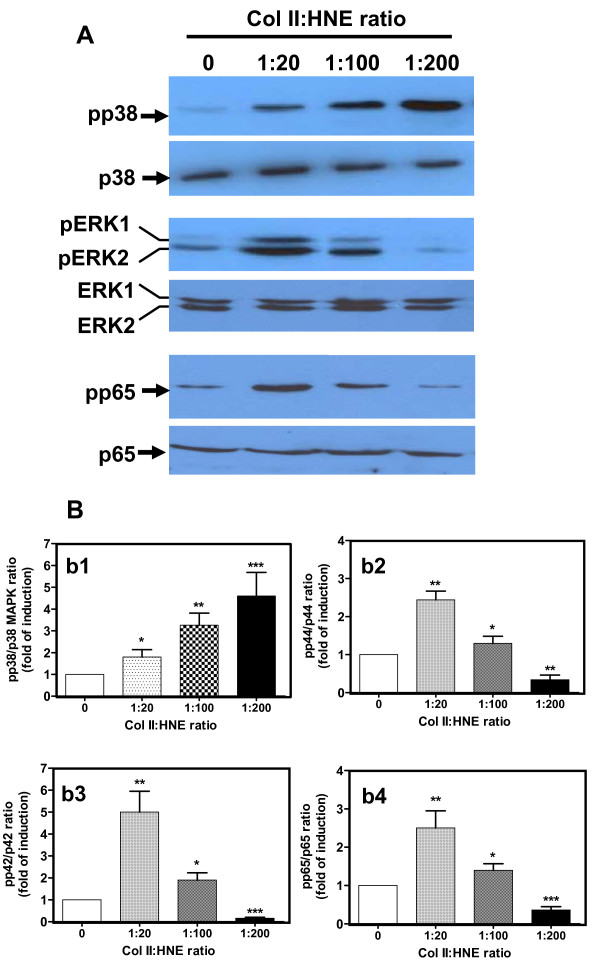
**Activation of MAPK and NF-κB pathways by HNE-modified Col II in human OA chondrocytes**. Cellular extracts from incubated chondrocytes (see legend 1) were subjected to assess total and phospho-p38 MAPK, ERK^1 ^(p44), ERK^2 ^(p42) and NF-κB-p65 by **(a) **Western blot analysis using specific antibodies or by **(b) **ELISA procedure using commercial kit. (b1) p38 MAPK, (b2) ERK^1^, (b3) ERK^2^, (b4) NF-κB-p65. ANOVA tests and Bonferroni's multiple comparison were performed to compare each condition. The data are means ± SEM of *n *= 4. *P < 0.05, **P < 0.01, ***P < 0.001.

To gain insights into the signalling pathways activated during interactions between chondrocytes and HNE-modified Col II, we examined the HNE-induced phosphorylation patterns of MAPKs over extended time periods by Western blotting and ELISA. As shown in Figure 5a, b, our data indicate that p38 MAPK, ERK^1/2 ^and NF-κB-p65 phosphorylation levels rose within 48 h of stimulation and depended on Col II alkylation level. p38 MAPK activation peaked when Col II was modified by HNE at 1:200 MR (Figure 5a, b.b1). In contrast, ERK^1/2 ^(Figure 5a, b.b2, b.b3) and NF-κB-p65 (Figure 5a, b.b4) activation peaked when Col II was exposed to HNE at a 1:20 MR HNE and declined gradually thereafter when Col II was modified by HNE at a 1:100 or 1:200 MR. However, our results disclosed that interactions between cells and HNE-modified Col II evoked weak activation of c-Jun N-terminal kinase 1/2 (JNK^1/2^) (data not included).

### CAR prevents HNE-modified Col II-induced chondrocyte functions

**Figure 6:**
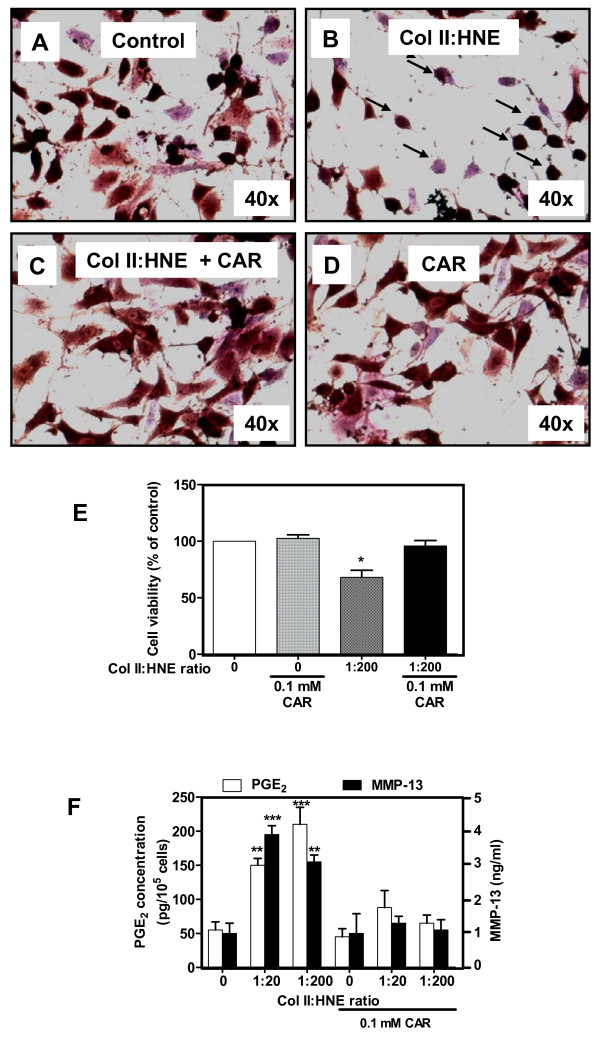
**Carnosine prevents HNE-modified Coll II-induced changes in chondrocytes phenotype and function**. A total of 0.1 mM carnosine was added to HNE-modified Col II-coated plates before cells seeding. Cell morphology of human OA chondrocytes was analysed by phase-contrast microscopy. Cells were incubated in **(a) **untreated Col II, **(b) **HNE-treated Col II at a 1:200 MR, **(c) **HNE-treated Col II at a 1:200 MR + 0.1 mM CAR, **(d) **0.1 mM CAR-treated Col II. **(e) **Cell viability of chondrocytes was evaluated by MTT assay. **(f) **Culture media from cultured cells was used to determine PGE_2 _and MMP-13 levels. ANOVA tests and Bonferroni's multiple comparison were performed to compare each condition. The data are means ± SEM of *n *= 4. *P < 0.05, **P < 0.01, ***P < 0.001.

To establish whether HNE-modified Col II induced changes in cell morphology and function could be potentially reversed by CAR, an HNE-trapping drug, 0.1 mM CAR was added to HNE-modified, Col II-coated plates one hour before cell seeding, and different factors were investigated. As seen in Figure 6, CAR prevented changes in cell morphology (Figure 6c) when compared to the untreated Col II (Figure 6a) and HNE-modified Col II at a 1:200 MR (Figure 6b). CAR alone had no effect on cell viability (Figure 6d). Interestingly, we observed that cell mortality as well as PGE_2 _and MMP-13 release were abolished by CAR (Figure 6e, f) when cells were incubated with HNE-modified Col II. These data indicated that CAR prevented changes in chondrocyte phenotype and function by establishing normal interactions between the cells and their surrounding matrix.

## Discussion

The main purpose of this study was to investigate interactions between human OA chondrocytes and HNE-modified Col II. When seeded in HNE-treated Coll II, significant amendment of chondrocyte morphology, from the typical chondrocyte-like polygon shape to rounded, semi-detached cells, was observed; mainly when Col II was modified by HNE at a 1:200 MR (Col II:HNE). Since morphological changes in cell shape are consistent with the induction of cell death, we studied cell viability and extrinsic caspase-3 activity. Our data disclosed that changes in cell morphology were accompanied by increased cell mortality, caspase-3 activation and loss of cell phenotype, as determined by Col II expression. Several authors have reported that fibroblast and osteoblast interactions with MDA-collagen adducts or advanced glycation end product (AGE)-modified collagen affected both the morphology and proliferation of these cells, leading to reduced cell adhesion, migration and viability [17,18]. It has been proposed that changes in both the physical properties and charge profiles of protein are particularly important for the attachment of cells to collagenous basement membranes. To determine that chondrocyte morphology and phenotype were closely correlated with cell-matrix interactions through adhesion molecules, we first investigated ICAM-1 and α1β1 integrin expression. Our results clearly showed that interactions between HNE-modified Coll II at a 1:20 MR and chondrocytes, probably through α1β1 receptors, significantly increased its own expression, and was dramatically decreased thereafter when Col II was modified by HNE at a 200 MR. However, ICAM-1 expression was significantly enhanced by HNE-treated Col II at a 1:20 MR and then declined to the control level. Since Col II is a ligand for α1β1 integrin, we suggest that the increment of ICAM-1 expression is α1β1-dependent. The involvement of α1β1 integrin in ICAM-1 expression was proposed by Nakayamada *et al. *[19], who postulated that the interaction of β1 integrin with the ECM heightens ICAM-1 expression in synovial cells. In OA, integrin and ICAM-1 receptors play a critical role in maintaining cartilage homeostasis [20]. High ICAM-1 and α1β1 integrin expression levels in the early stages of OA are indicated by several reports in humans and animals [20-22]. In adult joints, increased of β1 integrin was reported in osteoarthritic monkey cartilage compared to normal cartilage [22] and in human OA samples at minimally damaged locations compared to areas with more severe lesions [23]. Knee joints of α1β1-null mice display precocious proteoglycans loss, cartilage erosion associated with increased MMP-2 and MMP-3 expression, and synovial hyperplasia [24,25].

Then after, we have used immunoprecipitation method to examine the possibility that FAK participates in signaling from α1β1 integrins after cell adhesion. We provide evidence for formation of a α1β1 integrin-pFAK complex in cultured chondrocytes. Moreover, we demonstrate that the phosphorylated level of FAK decreased when cells were incubated with HNE-modified Col II at a 1:200 MR. These findings support the role of FAK as intermediates in α1β1 integrin-dependent signaling in chondrocytes adhering to HNE-Col II adducts. Our work, interpreted in the context of what is known about focal adhesion assembly, suggests that chondrocytes adhesion to HNE-Col II adducts by α1β1 integrins leads to recruitment of FAK. Intracellular signaling from the ECM is mediated through major cell-surface receptors called integrins, which serve as transmembrane links between the extracellular environment and focal adhesions within the cell [26]. Interaction of integrins with the ECM at these focal adhesions leads to recruitment of several signaling molecules such as paxillin, vinculin, talin and FAK [27,28] Interaction between β1 integrin and Col II in chondrocytes protects cells against apoptosis and mediates responses to external changes by maintaining the tissue composition and mechanical properties of articular cartilage [5].

Previous reports have demonstrated that ECM modification generates a panoply of signalling pathways implicated in various physiological and pathophysiological events [29]. To investigate this concept, we studied the possibility that HNE-modified Col II modulates catabolic and inflammatory responses known to be involved in OA through MAPK and NF-κB activation. Our data clearly showed that low alkylation of Col II strongly activates ERK^1/2 ^and NF-κB-p65 and slightly activates p38 MAPK. However, high alkylation of Col II dramatically inhibits ERK^1/2 ^and NF-κB-p65 and strongly stimulates p38 MAPK. Collectively, our findings suggest that NF-κB/ERK^1/2 ^and p38 kinase oppositely regulate cell viability and adhesion in the presence of HNE-Col II adducts. Integrins control cell adhesion, migration, and survival by activating complex signalling networks that involve MAPK members, such as ERK [30]. Previous studies with human chondrocytes have determined that activation of the β_1 _integrin and subsequent ERK^1/2 ^and NF-κB signalling pathways is extremely important for cell differentiation and survival, and the inhibition of which can induce apoptosis [31,32]. Stupack *et al. *[33] suggested that "integrin-mediated death" is elicited by the cytoplasmic domain of unligated β_1_-integrin, resulting in caspase-8 recruitment to the cell membrane. In contrast, Wei *et al. *[34] found that p38 MAPK activity in chondrocytes is essential for the induction of cell death. p38 MAPK forms a complex with caspase-8 and consequently attaches to the death-executing machinery.

Since OA is associated with cartilage degradation and synovium inflammation, we investigated the ability of HNE-Col II adducts to induce catabolic and inflammatory genes. Among them, MMP-13 and COX-2 assumed a crucial role. We found that interactions between chondrocytes and the HNE-modified matrix specifically evoked MMP-13 by low Col II alkylation and COX-2 by high Col II alkylation. These data, combined with those on signalling pathways, suggest that MMP-13 and COX-2 expression depend mainly on ERK^1/2 ^and p38 MAPK, respectively. It has been reported that Col I and fibronectin fragments induce MMP-13 expression in chondrocytes through ERK^1/2 ^signalling via α1β1 and αvβ3 integrin, respectively [5,35,36]. However, it has been observed that integrin-mediated adhesion to ECM proteins induces *de *COX-2 synthesis involving signalling through the p38 MAPK pathway [37]. The role of AGE-modified proteins in OA has been demonstrated in previous studies. It has been found that AGEs significantly stimulate a panoply of signalling pathways-mediating MMP-1, -3, and -13 expression [38-40]. These responses occur through receptors for AGEs, implicating those engaged in catabolic and inflammatory processes in OA.

Finally, we tested the hypothesis that CAR treatment is capable of inhibiting HNE-Col II adduct-induced changes in cell morphology and function. The data revealed that 0.1 mM CAR prevents changes in cell morphology and viability and blocked the production of factors known to be involved in OA, such as MMP-13 and PGE_2_. CAR exerts its effect in a dose-dependent manner with the maximum at 0.1 mM (data not shown). CAR was previously found to directly trap HNE [41]. By trapping HNE in stable covalent adducts, CAR can inhibit HNE-induced protein cross-linking. Therefore, it has the ability to displace bound HNE off the protein surface or to bind covalently into HNE-protein adducts. It is noteworthy that CAR can be covalently incorporated into glycated proteins, and consequently reduce protein carbonyl content [42]. In addition, CAR has been observed to reverse the physicochemical changes of proteins induced by glycation [43]. CAR, a dipeptide (b-alanyl-L-histidine), is considered to be a natural antioxidant and antiglycating agent that suppresses protein modifications by AGEs and LPO products [44,45]. It may play an important part as an anti-aging molecule, since it can delay senescence in cultured fibroblasts and reverse the senescent phenotype in cultured human cells [46]. Furthermore, it is useful in preventing several diseases, as beautifully depicted in a recent, dedicated review [47].

## Conclusions

**Figure 7:**
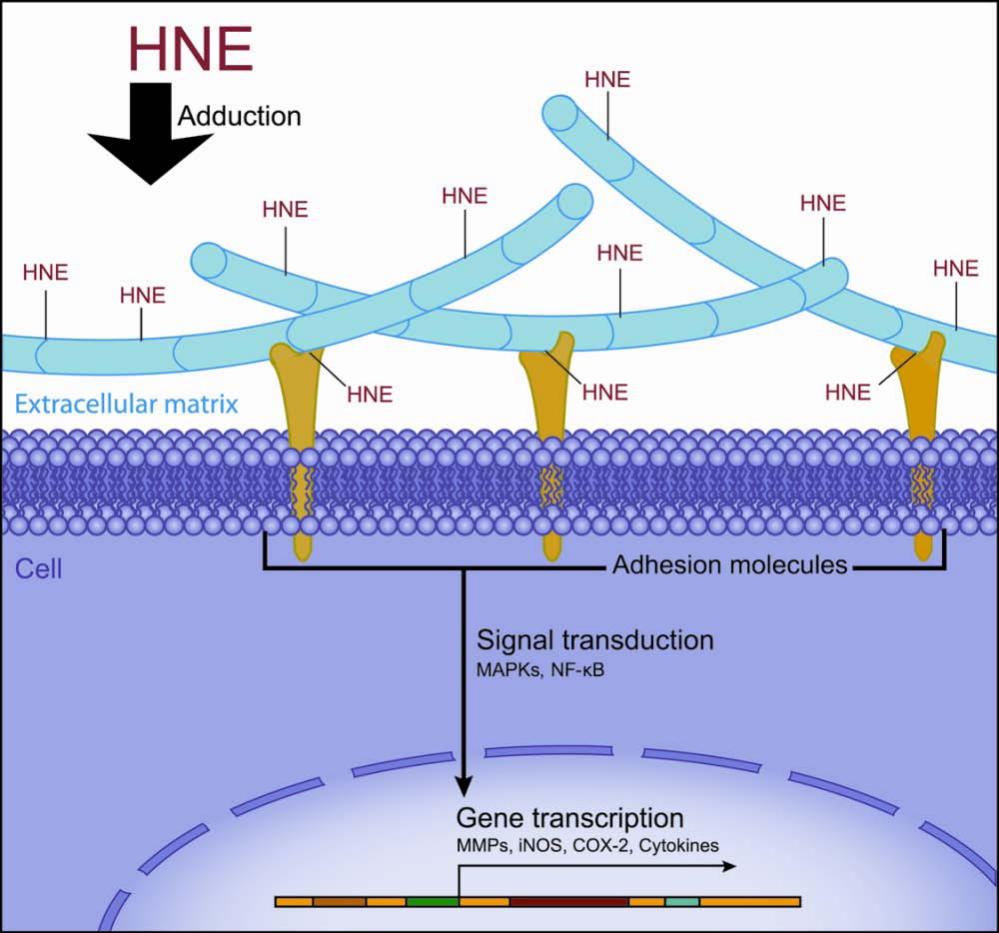
**Effect of HNE binding to extracellular matrix on cell function**. Overview of different signalling pathways target genes expression-induced by interactions between cell and HNE-modified matrix.

Collectively, we presented evidence that Col II modification by HNE is capable of inducing changes in chondrocyte phenotype and function. Our results clearly showed that interactions between chondrocytes and HNE-Col II adducts, probably via adhesion molecules, strongly evoking a panoply of signalling pathways that trigger cell adhesion and viability as well as MMP-13 and COX-2 expression, as illustrated in Figure 7. The fact that HNE-Col II adducts elicited cell death and catabolic and inflammatory responses suggested their involvement in OA. CAR has been found to prevent the effects of HNE-Col II adducts, although the mechanism of action has not yet been elucidated. Further research is required to understand the implication of Col II modification by HNE and other ECM components in OA as well as the potential of HNE-trapping drugs in OA.

## Abbreviations

CAR: carnosine; Col II: Type II collagen; COX-2: cyclooxygenase-2, FAK: focal adhesion kinase; HNE: hydroxynonenal; ICAM-1: intercellular signal-regulated molecule-1; LPO; lipid peroxidation; MAPK: mitogen-activated protein kinases; MMPs: matrix metalloproteinases; NF-κB: nuclear factor-kappa B; OA: osteoarthritis, PGE_2_: prostaglandin E2.

## Competing interests

The authors declare that they have no competing interests.

## Authors' contributions

REB performed the experimental study, contributed to preparation of the manuscript and undertook the statistical analysis. QS assisted in the experiments and in the isolation of chondrocytes from human cartilage. YM and LM performed confocal microscopy studies and MW performed flow cytometry analysis. JFC, HF, MDB, KM and JCF evaluated and interpreted the data and assisted with preparation of the manuscript. MB designed the study, supervised the project, evaluated and interpreted the data, and prepared the manuscript. All authors read and approved the final manuscript.

## Author information

Dr. M Benderdour is a research scholar of the FRSQ. J-F Côté holds a CIHR New Investigator award.
